# The prevalence of common CFTR gene mutations and polymorphisms in infertile Iranian men with very severe oligozoospermia

**DOI:** 10.25122/jml-2021-0261

**Published:** 2022-04

**Authors:** Leyla Jafari, Kyumars Safinejad, Mahboobeh Nasiri, Mansour Heidari, Massoud Houshmand

**Affiliations:** 1. Department of Biology, Arsanjan Branch, Islamic Azad University, Arsanjan, Iran; 2. Department of Biology, Borujerd Branch, Islamic Azad University, Borujerd, Iran; 3. Department of Medical Genetics, Tehran University of Medical Sciences (TUMS), Tehran, Iran; 4. Department of Medical Genetics, National Institute for Genetic Engineering and Biotechnology, Tehran, Iran

**Keywords:** CFTR gene, M470V polymorphism, very severe oligozoospermia, IVS8 poly T, N1303K, R117H, CF – Cystic fibrosis, CBAVD – Congenital bilateral absence of the vas deferens, ICSI – Intracytoplasmic sperm injection, ARMA – Amplification-refractory mutation system, ACECR – Academic Center for Education, Culture, and Research, EDTA – Ethylenediaminetetraacetic acid, SSCP – Single strand conformational polymorphism

## Abstract

Due to progress in infertility etiology, several genetic bases of infertility are revealed today. This study aimed to investigate the distribution of mutations in the CFTR gene, M470V polymorphism, and IVS8 poly T. Furthermore, we aimed to examine the hotspot exons (4, 7, 9, 10, 11, 20, and 21 exons) to find a new mutation in cystic fibrosis transmembrane conductance regulator (CFTR) gene among infertile Iranian men very severe oligozoospermia (<1 million sperm/mL ejaculate fluid). In the present case-control study, 200 very severe oligozoospermia (20–60s) and 200 fertile men (18–65s) were registered. Five common CFTR mutations were genotyped using the ARMS-PCR technique. The M470V polymorphism was checked out by real-time PCR, and poly T and exons were sequenced. The F508del was the most common (4.5%) CFTR gene mutation; G542X and W1282X were detected with 1.5% and 1%, respectively. N1303K and R117H were detected in 0.5% of cases. F508del was seen as a heterozygous compound with G542X in one patient and with W1282X in the other patient. Also, in the case of M470V polymorphism, there are differences between the case and control groups (p=0.013). Poly T assay showed statistical differences in some genotypes. The study showed no new mutation in the exons mentioned above. Our results shed light on the genetic basis of men with very severe oligozoospermia in the Iranian population, which will support therapy decisions among infertile men.

## Introduction

Infertility is described as the inability to get pregnant (conceive) after one year (or longer) of regular intercourse [1, 2]. It is estimated that 15% of couples are infertile, with half of all infertility cases involving men [3]. Despite all the known causes of male infertility, unexplained infertility remains unclear. Many factors contribute to male infertility, including congenital or acquired abnormalities of the genital tract, infections, endocrine disorders, malignancies, immune disorders, and genetic abnormalities. A US study of 1,430 patients identified the most common rare causes of infertility, including varicocele, idiopathic cause, obstruction, gynecological factor, cryptorchidism, immunology, ejaculatory dysfunction, testicular failure, drug/radiation effects, and disorder of the endocrine system [4]. However, despite recent technological and diagnostic advances, idiopathic infertility is a common cause and accounts for approximately 25% of all causes of infertility [5, 6]. In addition, many of the identifiable causes of male infertility are treatable or preventable, so it is important to have a clear understanding of the disorder. Genetic causes play a decisive role in the development of idiopathic azoospermia and severe oligozoospermia, so 30% of the men who refer to infertility treatment clinics have genetic abnormalities [7].

The CFTR gene is one of the genes confirmed to play a role in infertility. The CFTR gene mutations were observed in 85% of patients with CBAVD [8]. It should be noted that most men with cystic fibrosis are infertile due to CBAVD [9]. The CFTR gene is a member of the ATP-binding gene superfamily and is widely expressed in the apical membrane of secretory epithelial cells and the reproductive tissues that regulate the vas deferens [10]. The CFTR gene contains DNA of more than 180,000 base pairs (bp) and 27 exons and is located on the short arm of chromosome 7 [11, 12]. There are approximately more than 1,500 CFTR variants in the CFTR database. Considering the population distribution, more than 30 major mutations in CFTR have been identified. These mutations include ΔF508, IVS8-5T, R117H etc [1]. The ΔF508 mutation of the CFTR gene, which leads to the wrong folding of the CFTR protein, leads to the retention of the CFTR protein in the endoplasmic reticulum [13]. Alleles T9, T7, and T5 are three common forms of IVS8-Tn polymorphism that act as receptor sites for exon 9 splicing sites. In addition, the IVS8-T5 form is now referred to as a mutation rather than a polymorphism [14]. The alternation between arginine and histidine in the R117H mutation of the 117 exon 4 loci of the CFTR gene affects the pore characteristics and the CFTR channel gate [15]. There are new mutations, and polymorphisms of M470V and IVS8 poly T CFTR gene in men with very severe oligozoospermia referred to the infertility treatment center in Qom city, Iran.

## Material and Methods

**Figure 1.**
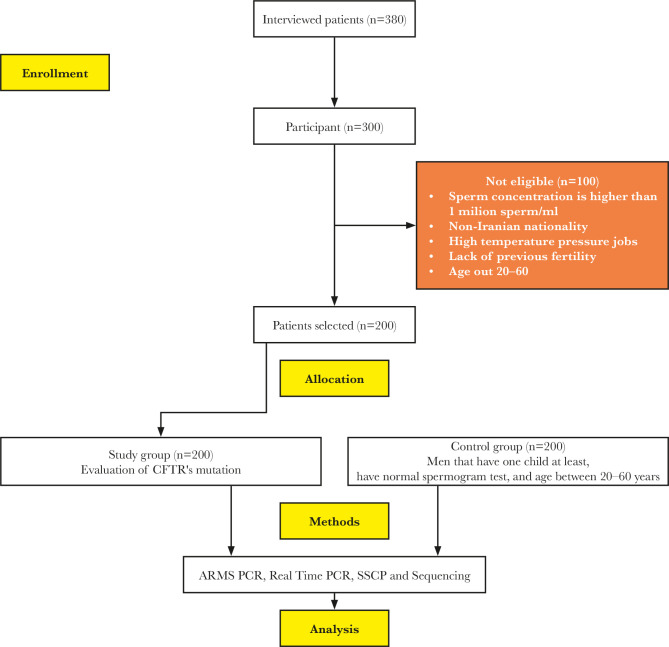
Study flowchart.

The present case-control study was performed on 200 infertile men aged 20 to 60 years with very severe oligozoospermia (case) and 200 healthy men aged 18 to 65 years (control). The diagram in Figure 1 shows the enrollment process and exclusion of individuals step by step in the study.

### DNA extraction and genotyping

5 ml of venous blood was taken from all participants and poured into tubes containing ethylene diamine tetrastic acid (EDTA). These blood samples were stored at -20°C for molecular testing. The genome was extracted from blood samples using a DNA extraction kit according to the manufacturer's instructions (Sinagen, Iran). The quality of the extracted DNA was evaluated using a nanodrop spectrophotometer.

### CFTR gene mutations identification

Amplification-Refractory Mutation System (ARMS-PCR) was used to determine the genotype of common mutations in the CFTR gene (F508del, G542X, N1303K, W1282X, and R117H). Appropriate primers were designed using primer3 software. Relevant specifications, such as sequence and band size, are shown in Table 1. Genotypes after electrophoresis based on different sizes of PCR products and gel staining using Safe Stain dye (Pishgam Company, Iran) were determined on agarose gel (Tables 2 and 3).

**Table 1.** Primers sequences and the amplified product sizes.

**Primers**	**Primer sequence (5' to 3')**	**Size (bp)**	**Primers**	**Primer sequence (5' to 3')**	**Size (bp)**
**ΔF508**	GACTTCACTTCTAATGATGATTATGGGAG GTATCTATATTCATCATAGGAAACACCAC GTATCTATATTCATCATAGGAAACACCAT	160 157	Exon 4	TCACATATGGTATGACCCTC TTGTACCAGCTCACTACCTA	438
**G542X**	GACTTCACTTCTAATGATGATTATGGGAG ACTCAGTGTGATTCCACCTTCTAC CACTCAGTGTGATTCCACCTTCTCA	256 257	Exon 7	AGACCATGCTCAGATCTTCCAT GCAAAGTTCATTAGAACTGATC	410
**R117H**	CACATATGGTATGACCCTCTATATAAACTC CCTATGCCTAGATAAATCGCGATAGAAC' CCTATGCCTAGATAAATCGCGATAGAAT	237 237	Exon 9	CATAAAACAAGCATCTATTG AGAGACATGGACACCAAATT	322
**N1303K**	CTCAATTTCTTTATTCTAAAGACATTGG GATCACTCCACTGTTCATAGGGATCCAAG GATCACTCCACTGTTCATAGGGATCCAAC	328 328	Exon 10	GCAGAGTACCTGAAACAGGA CATTCACAGTAGCTTACCCA	491
**W1282X**	CCCATCACTTTTACCTTATAGGTGGGCCTC CCTGTGGTATCACTCCAAAGGCTTTCCAC CCTGTGGTATCACTCCAAAGGCTTTCCAT	178 178	Exon 11	CAACTGTGGTTAAAGCAATAGTGT GCACAGATTCTGAGTAACCATAAT	425
**M470V**	CTTCTGCTTAGGATGATAATTGG GCTTTGATGACGCTTCTGTA	Probe for Nucleotide A: cttctaatg (A) tg Nucleotide G: tctaatg (G) tga	Exon 20	GGTCAGGATTGAAAGTGTGCA CTATGAGAAAACTGCACTGGA	471
**IVS8**	5T forward: GTGTGTGTGTGTGTGTGTTGTT 7T forward: GTGTGTGTGTGTGTGTTTTGTT Reverse: GACATGGACACCAAATTAAG	-	Exon 21	AATGTTCACAAGGGACTCCA CAAAAGTACCTGTTGCTCCA	477

**Table 2.** PCR conditions to amplify ΔF508.

**Step**	**Temperature (°C)**	**Time**	**Cycle**
**Initial Denaturation**	95	3 min	1
**Denaturation**	93	35 sec	35
**Annealing**	53	40 sec
**Extension**	72	35 sec
**Final extension**	72	5 min	1

**Table 3.** PCR conditions to amplify G542X, R117H, N1303K and W1282X.

**Step**	**Temperature (°C)**	**Time**	**Cycle**
**Initial Denaturation**	95	5 min	1
**Denaturation**	95	30 sec	35
**Annealing**	50	60 sec	
**Extension**	72	60 sec	
**Final extension**	72	10 min	1

### Real-time PCR to detect M470V mutation

Detection of M470V polymorphism was performed using two special probes and a Real-Time PCR test. Primers, probes, PCR conditions, and components are presented in Tables 1 and 4.

**Table 4.** PCR conditions to amplify M470V.

**Step**	**Temperature (°C)**	**Time**	**Cycle**
**Initial Denaturation**	95	30 sec	1
**Denaturation**	95	5 sec	50
**Annealing**	57	15 sec	
**Extension**	72	34 sec	
**Final extension**	72	5 min	1

### Poly T in IVS8

Considering the intron 8 genotype associated with the poly T sequence, two primers were selected for T5 and T7 sequences (Table 1) [16]. 20 μl of PCR product (260–264bp) was digested with 5 U HpaI enzyme and incubated overnight at 37°C. After digestion, the products were electrophoresed on 8% acrylamide gel at 220V for 2.5 hours (Table 5).

**Table 5.** PCR conditions to amplify IVS8.

**Step**	**Temperature (°C)**	**Time**	**Cycle**
**Initial Denaturation**	95	30 sec	1
**Denaturation**	95	5 sec	35
**Annealing**	57	15 sec	
**Extension**	72	34 sec	
**Final extension**	72	5 min	1
**Initial Denaturation**	95	30 sec	1
**Denaturation**	64	40 sec	35
**Annealing**	70	90 sec	
**Extension**	72	180 sec	1

### CFTR gene new mutations assay

The single-strand conformational polymorphism (SSCP) technique was performed on samples that did not have mutations. Exons 4, 7, 9, 10, 11, 20, and 21 were amplified using the primers shown in Table 1. After performing SSCP, bands with different sizes than the positive control were selected and sequenced. This means that the rest of the bands, which were the same size, had no mutation (Tables 6 and 7).

**Table 6.** PCR conditions to amplify Exons 4, 9 and 10.

**Step**	**Temperature (°C)**	**Time**	**Cycle**
**Initial Denaturation**	95	3 min	1
**Denaturation**	95	30 sec	35
**Annealing**	56	40 sec	
**Extension**	72	30 sec	
**Final extension**	72	5 min	1

**Table 7.** PCR condition to amplify Exons 7, 11, 20 and 21.

**Step**	**Temperature (°C)**	**Time**	**Cycle**
**Initial Denaturation**	95	5 min	1
**Denaturation**	95	30 sec	35
**Annealing**	52	60 sec	
**Extension**	72	60 sec	
**Final extension**	72	10 min	1

### Statistical analyses

The statistical analyses were performed using SPSS statistical software (version 16.0, SPSS Inc., Chicago, IL, USA). The distribution of mutations in the patient and control groups was expressed as the number and frequency (percentage).

## Results

### CFTR gene mutations

**Figure 2.**
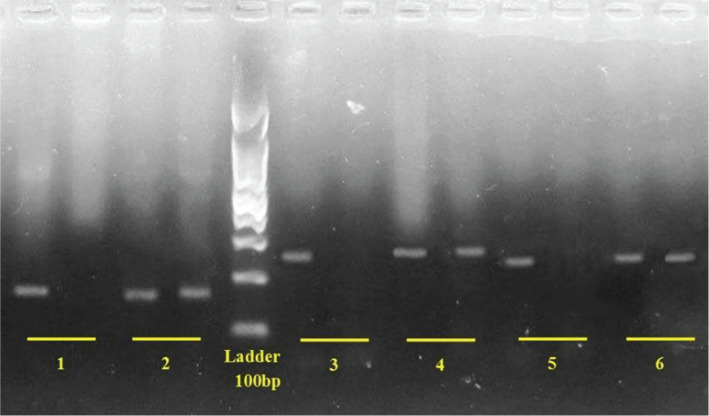
CFTR gene mutations genotyping on 2% agarose gel electrophoresis; 1. W1282X normal (178bp), 2. W1282X heterozygote (178bp) 3. G542X normal (257bp), 4. G542X heterozygote (256bp), 5. R117H normal (237 bp), 6. R117H heterozygous (236 bp).

**Figure 3.**
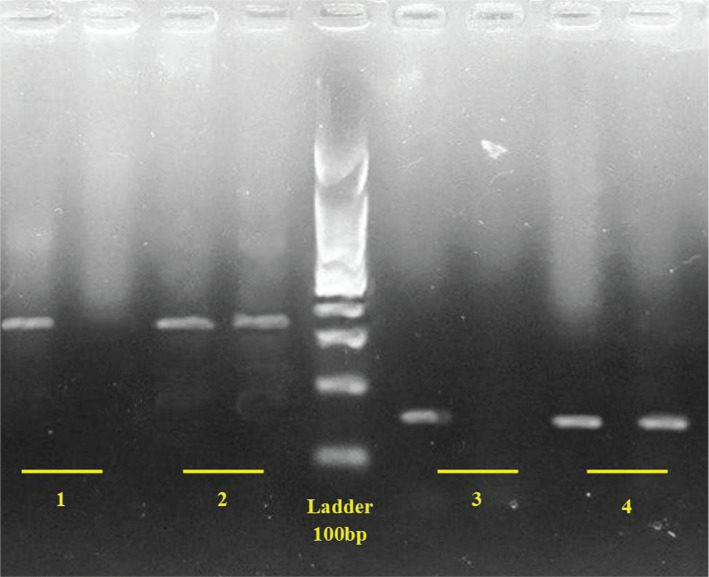
CFTR gene mutations genotyping on 2% agarose gel electrophoresis; 1. N1303K normal (328bp), 2. N1303K heterozygote (328bp), 3. F508del normal (160bp), 4. F508del heterozygote (157bp).

The most common mutation was F508del, which accounted for 4.5% of cases. The two nonsense mutations, G542X and W1282X with 1.5 and 1%, respectively, were the second and third most common mutations in this gene. N1303K and R117H mutations were observed in equal proportions (0.5%) in the studied population (Tables 8 and 9). Some of these mutations are shown in Figures 2 and 3. 

**Table 8.** CFTR gene mutations distribution.

**Mutations**	**Protein change**	**cDNA position**	**Mutant cases (Heterozygote)**
**F508del**	p.Phe508del	c.1521_1523delCTT	9 (4.5%)
**G542X**	p.Gly542X	c.1624G>T	3 (1.5%)
**W1282X**	p.Try1282X	c.3846G>A	2 (1%)
**N1303K**	p.Asn1303Lys	c.3909C>G	1 (0.5%)
**R117H**	p.Arg117His	c.350G>A	1 (0.5%)

**Table 9.** The simultaneous occurrence of genetic changes in Yq or CFTR and AR genes.

**F508del**	**G542X**	**R117H**	**W1282X**	**number**	**Frequency (%)**
√	√			1	0.5
√			√	1	0.5

### Real-Time PCR

**Figure 4.**
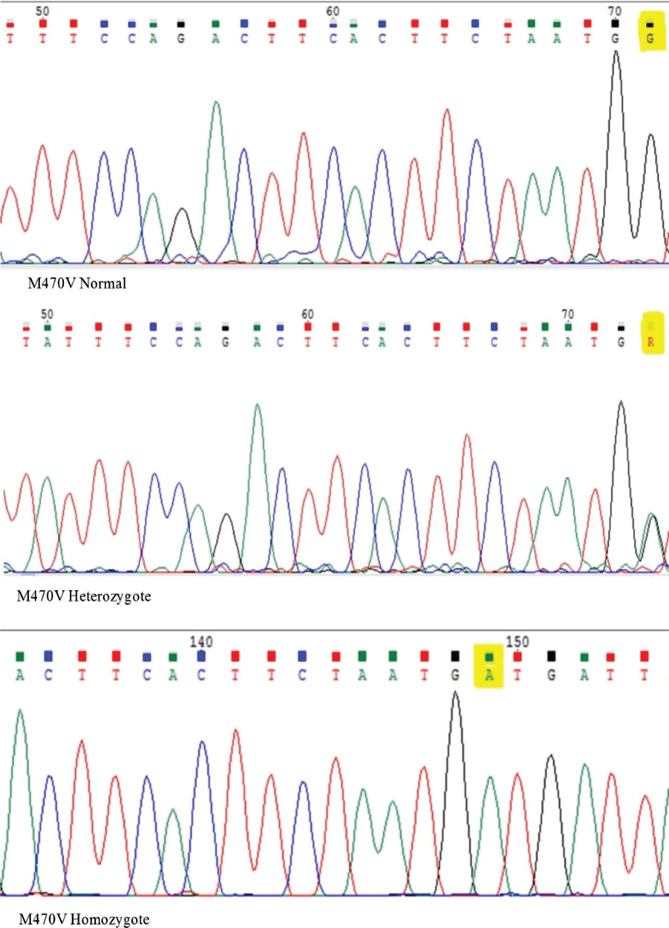
Sequencing of M470V in homozygote and heterozygote status.

The polymorphism results by G and A probes showed that 79 patients (39%) had heterozygous GA polymorphism and 36 patients (18%) had AA mutant homozygosity. The prevalence and related sequences are shown in Table 10 and Figure 4. Chi-square analysis showed a significant difference between the two populations in this regard (p=0.013).

**Table 10.** M470V polymorphism prevalence in case and control.

**Sample**	**Mutations**	**Protein change**	**cDNA position**	**Normal (Homozygote)**	**Mutant cases (Heterozygote)**	**Mutant cases (Homozygote)**
**Case**	M470V	p.Val470Met	c.1408G>A	85 (43%)	79 (39%)	36 (18%)
**Control**	M470V	p.Val470Met	c.1408G>A	114 (57%)	64 (32%)	22 (11%)

### Poly T assay

**Figure 5.**
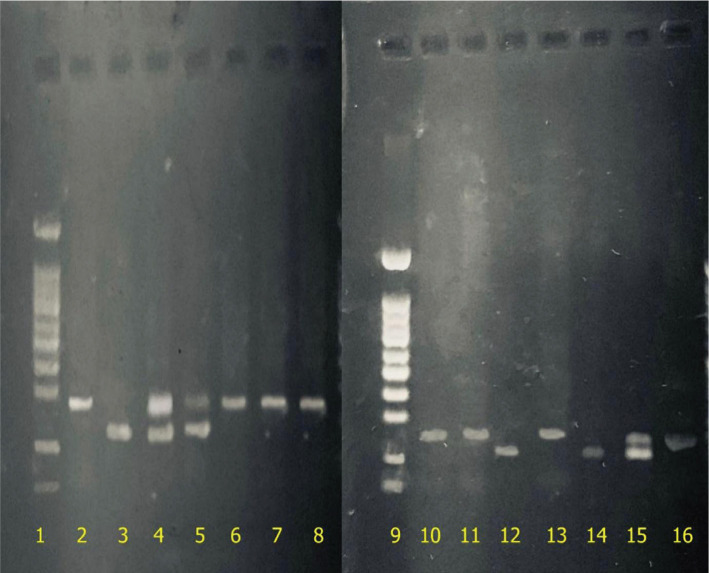
HpaI digestion results on ethidium-bromide–stained polyacrylamide gel. 1 &9 Marker 100bp. 2 &10 Uncut product, 3 &11 5T5T, 4 &12 5T7T, 5 &13 5T9T, 6 &14 7T7T, 7 &15 7T9T and 8 &16 9T9T.

The results obtained after amplification by PCR and digestion by the HpaI enzyme are shown in Figure 5. Among all genotypes, 5T/5T, 5T/7T, and 7T/7T genotypes had statistically significant differences between case and control groups (Table 11). 

**Table 11.** Poly T genotype distribution in case and control.

**Sample**	**5T/5T n (%)**	**5T/7T n (%)**	**5T/9T n (%)**	**7T/7T n (%)**	**7T/9T n (%)**	**9T/9T n (%)**
**Case**	22 (11%)	66 (33%)	5 (2.5%)	104 (52%)	2 (1%)	1 (0.5%)
**Control**	4 (2%)	16 (8%)	2 (1%)	177 (88.5)	1 (0.5%)	0 (0%)
**P value**	<0.01	<0.01	0.069	<0.01	0.169	0.073

### New mutation assay

**Figure 6.**
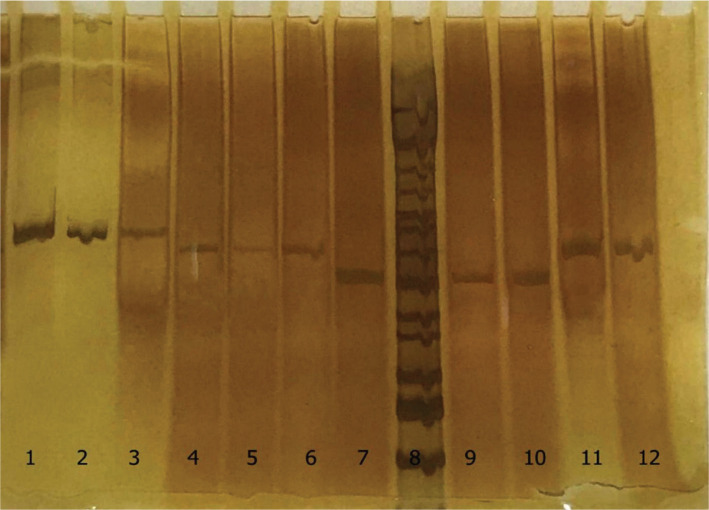
PCR product of hotspot exons. 1-3 exon10 (491bp), 4-6 exon 11(425bp), 7 and 9-10 exon 9 (322bp), 11-12 exon 4 (438bp), lane 8 marker 50bp.

**Figure 7.**
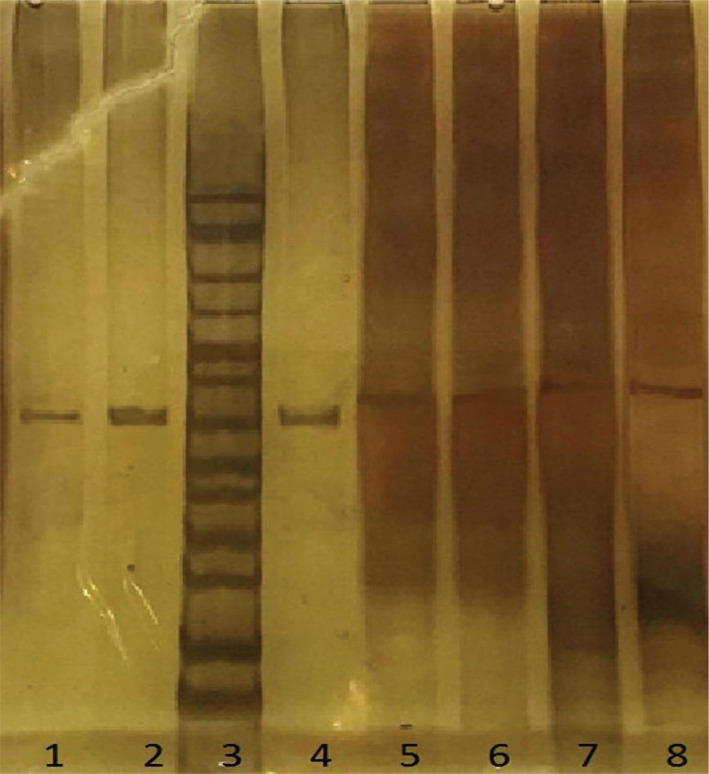
PCR product of hotspot exons. 1-2 and 4 exon7 (410bp), 5-6 exon 20 (471bp), 7-8 exon 21(477bp), lane 3 marker 50bp.

The sequencing examination of sample results related to hot spot exons did not show any mutation in these areas. First, the PCR-SSCP technique was performed on the samples. That is, after amplification of the mentioned exons, the PCR products were loaded on 8% acrylamide gel to find the difference between the motion of the PCR product compared to the positive control. No significant differences were found in band lengths, and a number of samples were sequenced to ensure that mutations were found, and the results without their new mutations are shown in Figures 6 and 7.

## Discussion

The CFTR gene is expressed throughout the reproductive system. On the other hand, an important role for this channel in sperm function has been identified by interfering with HCO-3 secretion and its effect on sperm fertilization capacity [17]. This channel, which is present in the membrane of human sperm cells, affects not only sperm function but also male fertility. In addition to decreased sperm motility, decreased fertility was observed in mice with CFTR deficiency [18]. So far, more than 1,400 different mutations have been identified in the CFTR gene. The most common mutation in the CFTR gene is the deletion of a single G nucleotide, which results in the deletion of the amino acid phenylalanine at the 508 codon position. This mutation is responsible for 66% of CFTR gene mutations that differ in different geographical locations and ethnic groups [19]. Although the genetic correlation between CFTR gene mutations and CBAVD-induced male infertility has been well studied, it has recently been established that CFTR gene mutations are involved in other forms of male infertility in addition to the CBAVD phenotype. However, the association between changes in sperm parameters and the CFTR gene appears to be weak and remains largely unknown [20, 21]. Our study helps to better identify this association, especially the association between severe oligozoospermia and CFTR gene changes, and shows more realistic results because of the large number of patients and controls. Due to the rarity of this type of patient, this study lasted for more than 2 years without interruption. There is evidence of the CFTR protein involvement in reducing sperm cytoplasmic volume during spermatogenesis in a study on rat testicular tissue in which CFTR gene mRNA was restricted to precursor round spermatozoa and primary cells which form the primary part of the epididymis of rodents and human [22].

Although various studies on the frequency of CFTR mutations in infertile men without CBAVD reported conflicting results, in some groups, increasing the frequency of the CFTR mutations is associated with decreasing sperm quality [23], idiopathic male infertility [24] and cryptozoospermia [25]. In contrast, some studies did not observe an increase in the frequency of the CFTR mutations in men with non-obstructive azoospermia or oligoasthenoteratozoospermia [26]. However, the small number of people in that study may be the cause of their conflicting results. The need to screen for CFTR mutations in infertile men, such as before intracytoplasmic sperm injection (ICSI), has not been fully explored yet. The present study helps resolve this contradiction, especially as more people have been studied. However, this could be regarded as a new study since instead of severe oligozoospermia (sperm count less than 5 million per millimeter of semen), men with very severe oligozoospermia (sperm count less than 1 million per millimeter of semen) were involved. 

In general, the total frequency of the CFTR gene mutations was 8%, which corresponds with Schulz *et al.* results, where 7.69% of patients with severe oligozoospermia had a CFTR mutation [27]. According to Sharma *et al.* (2014), non-obstructive azoospermia was about 11%, and in people with spermatogenesis defects, it was about 7%. On the other hand, it was reported that the homozygous mutation of the T5 allele in this population is higher than in other populations [28], which reinforces the present study results. The present study also showed that mutation detection using conventional and low-cost methods such as ARMS-PCR and PCR-SSCP and its confirmation by sequencing could easily detect the CFTR gene mutations. Due to mechanical life and increased stress and the potential for CFTR mutations, increasing age of marriage, especially in men, constant division of mitosis and meiosis in sexual gonads throughout life, men transmit new gene mutations to the next generation. Genetic testing of the CFTR gene in men with very severe oligozoospermia can be helpful in several ways. First, by identifying this mutation in men and following up and trying to diagnose this mutation in women, cystic fibrosis (CF) disease can be prevented with the help of the preimplantation genetic diagnosis (PGD) technique. At least with the same PGD technique, the transfer of CFTR gene mutation to the next generation can be prevented.

If further studies reveal a link between the CFTR gene mutation and very severe oligozoospermia, we can prevent the transmission of this mutation to the next generation.

Before infertility treatment of the men with severe oligozoospermia, genetic counseling and laboratory testing of CFTR gene mutations should be performed to prevent transmission of the relevant gene mutation or CF disease to the next generation. Studies are recommended to examine all exons of the CFTR gene in patients without a common mutation.

## Conclusion

Our study indicates that ICSI in couples with very severe oligozoospermia can lead to an increase in children at risk for cystic fibrosis if both parties carry the CFTR gene mutation. Genetic testing and counseling before ICSI are recommended for these couples.

## Acknowledgements

### Conflict of interest

The authors declare no conflict of interest.

### Ethical approval

This study was approved by the Ethics Committee of the Center for Education, Culture and Advanced Academic Research (ACECR) (no. IR.IAU.QOM.REC. 1399.014) in our local department.

### Consent to participate

All participants received and signed informed consent before participating in the study.

### Authorship

KS is the general coordinator who created the project. KS and MN designed the research study, organized the project, and performed the research. LJ performed PCR-based experiments and analysis of DNA sequencing data. MH and MHM analyzed the data and performed the statistical analysis. KS, LJ, and MN wrote a comprehensive literature review. KS and LJ wrote the paper. All authors approved the final version of the manuscript and submission of the manuscript.
